# Skeletal Muscle miRNA Patterns in High-Altitude Trekkers: Exploratory Identification of Molecular Signatures of Cellular and Endocrine Adaptation

**DOI:** 10.3390/biom16050668

**Published:** 2026-05-01

**Authors:** Tiziana Pietrangelo, Paolo Cocci, Danilo Bondi, Vittore Verratti, Carmen Santangelo, Lorenzo Marramiero, Francesco Alessandro Palermo

**Affiliations:** 1 Department of Neuroscience, Imaging and Clinical Sciences, University “G. d’Annunzio” of Chieti-Pescara, 66100 Chieti, Italy; tiziana.pietrangelo@unich.it (T.P.); danilo.bondi@unich.it (D.B.); carmen.santangelo@unich.it (C.S.); lorenzo.marramiero@unich.it (L.M.); 2 School of Biosciences and Veterinary Medicine, University of Camerino, 62032 Camerino, Italy; paolo.cocci@unicam.it; 3 Department of Science, University “G. d’Annunzio” of Chieti-Pescara, 66100 Chieti, Italy; vittore.verratti@unich.it; 4 CIBIO-Department of Cellular, Computational and Integrative Biology, University of Trento, 38123 Trento, Italy

**Keywords:** high-altitude hypoxia, skeletal muscle, microRNA (miRNA), biomarkers, extreme environments

## Abstract

Exposure to high-altitude hypoxia leads to complex physiological and molecular adaptations, particularly in skeletal muscle. MicroRNAs (miRNAs), including muscle-enriched (myomiRNAs) and hypoxia-responsive (hypoxamiRNAs), play critical roles in regulating these responses. We investigated miRNA expression changes in the skeletal muscle of healthy, non-smoking Italian adults (mean age 36.7 ± 12.4 years) participating in the Himalayan expedition “Lobuche Peak—Pyramid Exploration & Physiology” conducted in the Sagaramāthā (Mount Everest) National Park, Nepal. The peak overnight stay altitude was ≈5000 m at the Pyramid International Laboratory—Observatory. Muscle biopsies were taken before and after the expedition from Vastus lateralis, at one-third of the distance from the upper margin of the rotula to the anterior superior iliac spine. Small RNA sequencing was used to profile differentially expressed miRNAs. Several miRNAs were differentially expressed (exploratory analysis), suggesting potential involvement in hypoxia-related adaptation. These encompass both canonical myomiRNAs (e.g., miR-206, miR-486-5p) and hypoxamiRNAs (e.g., miR-378a-5p, miR-199a-3p, let-7b-5p). In enrichment analysis, we found several connections between miRNAs and pathways that may play a role in physiological regeneration or differentiation in muscle cells. Among functions, focal adhesion (*p*-value = 0.001), regulation of actin cytoskeleton (*p*-value = 0.026), Rap-1 (*p*-value = 0.007), cAMP (*p*-value = 0.017), MAPK (*p*-value = 0.019), and Hippo (*p*-value = <0.001) signaling pathways were predicted to be the most targeted. These findings provide preliminary insights into physiological adaptation, requiring confirmation in larger and controlled cohorts.

## 1. Introduction

Exposure to high-altitude environments represents a potent physiological stressor due to the progressive decline in atmospheric oxygen availability with increasing elevation. This hypoxic condition triggers a series of adaptive responses in the human body aimed at maintaining oxygen supply. Among the various tissues affected, skeletal muscle is particularly sensitive to oxygen deprivation, exhibiting structural and functional changes such as reduced oxidative capacity and decreased aerobic performance. Paradoxically, despite these negative effects, hypoxia also serves as a physiological stimulus that can promote beneficial adaptations in skeletal muscle, especially when combined with physical activity [1]. At elevations above 3000 m, the drop of inspired partial pressure of oxygen induces ventilatory acclimatization and activates both hypoxia-inducible factor (HIF)-dependent and HIF-independent signaling pathways [2,3]. These pathways regulate cellular responses mediating angiogenesis, glycolytic metabolism, erythropoiesis, and apoptosis, thereby enabling tissue survival and function [4]. In skeletal muscle, acute and chronic hypoxia can modulate signaling cascades such as PI3K-AKT, mTOR, MAPK, and Notch-Wnt, finally influencing satellite cell activation, fiber-type switching, and mitochondrial metabolism [5,6].

An increasing number of studies have highlighted the pivotal role of microRNAs (miRNAs), small non-coding RNAs that regulate gene expression at the post-transcriptional level, in coordinating these adaptive responses. Specific miRNAs, known as hypoxamiRNAs, are differentially expressed under hypoxic conditions and act as fine-tuners of hypoxia-responsive genes [7,8]. In skeletal muscle, a subset of miRNAs termed myomiRNAs, including miR-1, miR-133, miR-206, and miR-486, play essential roles in muscle development, regeneration, and metabolic regulation [9,10,11]. These miRNAs are also implicated in the modulation of pathways influenced by hypoxia, making them potential biomarkers for oxygen-related stress and muscle adaptation. Beyond environmental physiology, the study of hypoxia-induced miRNA modulation may also have broader implications for conditions characterized by impaired oxygen delivery, such as aging-related muscle decline, ischemic disorders, and chronic diseases, where hypoxia-inducible pathways are activated but may lead to maladaptive responses. Despite these insights, few studies have directly investigated how prolonged exposure to high-altitude hypoxia affects miRNA expression in human skeletal muscle. Understanding the molecular basis of muscle adaptation to environmental hypoxia could provide new tools for monitoring acclimatization, optimizing athletic performance, and identifying individuals at risk for high-altitude illnesses.

This study aimed to exploratively assess (pilot design) miRNA expression changes in human skeletal muscle following high-altitude exposure, acknowledging the limited sample size and exploratory scope. We focused on identifying muscle-enriched and hypoxia-responsive miRNAs modulated by these combined stressors and on exploring the biological pathways potentially regulated by differentially expressed miRNAs to gain insight into muscle adaptation mechanisms. Overall, this work contributes to the establishment of a reference dataset for evaluating miRNA expression patterns and their regulatory networks during prolonged physical activity under extreme environmental conditions, such as hypoxia.

## 2. Materials and Methods

### 2.1. Altitude Expedition and Study Design

**Figure 1:**
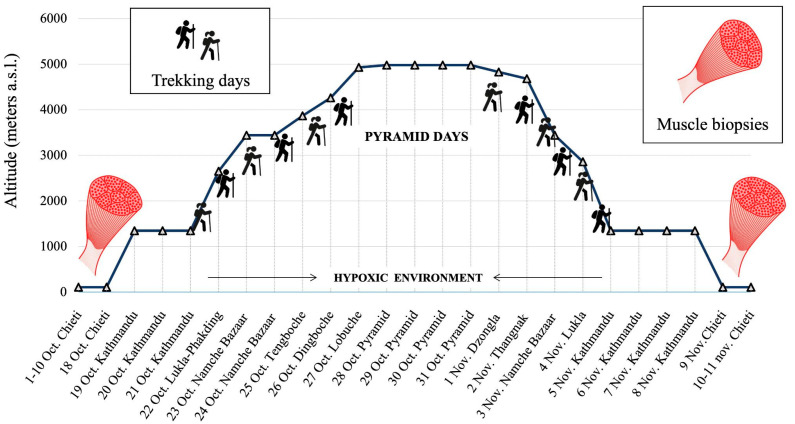
Study design, altimetric plan and time course of the trekking in the framework of the “Lobuche Peak—Pyramid Exploration & Physiology” Himalayan expedition.

The Himalayan expedition “Lobuche Peak—Pyramid Exploration & Physiology” conducted in the Sagaramāthā (Mount Everest) National Park, Nepal, from 19 October to 9 November 2022, included 21 healthy adults (Figure 1) [12,13]. Of these, six participants (3 males and 3 females, aged 36.7 ± 12.4 years, range: 25–54 years; BMI 23.1 ± 3.02 kg/m^2^, range: 18.8–27.2 kg/m^2^) were involved in the miRNOME assessment project. Additional demographic and lifestyle information was collected for all participants, including general physical activity level, occupational background, and medical history. All subjects were non-smokers and reported no regular use of alcohol or recreational drugs. No participant had a history of recent musculoskeletal injury or chronic disease affecting skeletal muscle function. All individuals were recreationally active but not professional athletes. Due to the limited sample size and privacy considerations, detailed individual-level data are not reported; however, the cohort can be considered homogeneous in terms of general health status and lifestyle. The selection of the six participants from the original 21 was made for logistical constraints about the expedition in extreme environments and individual logistical reasons, i.e., availability of individuals for biopsies before and after the expedition, quality of samples for subsequent processing, exclusion of any muscle impairment or injuries, representativeness of the group, and gender balance. During the ascent, acetazolamide (250 mg/day) was administered to all participants as standard prophylaxis against acute mountain sickness. This carbonic anhydrase inhibitor may influence acid–base balance, ventilation, and potentially skeletal muscle physiology, and is therefore considered a relevant confounding factor in the interpretation of molecular data. The peak overnight stay altitude was ≈5000 m at the Pyramid International Laboratory—Observatory. In studies conducted under extreme environmental conditions, such as high-altitude settings, logistical and operational constraints frequently necessitate the use of relatively small but rigorously characterized cohorts; nonetheless, such designs have consistently proven informative in investigations of adaptation to hypoxic stress [13,14].

### 2.2. Muscle Sampling and Satellite Cells Isolation

Muscle biopsies were taken before (in the previous 10 days) and after the expedition (from 5 to 20 h after returning to Italy); the timeline of exposure to different altitudes is shown in Figure 1. The timing of post-expedition biopsies may have allowed partial re-adaptation processes, which should be considered when interpreting hypoxia-related molecular responses. Biopsies were taken from Vastus lateralis, at one-third of the distance from the upper margin of the rotula to the anterior superior iliac spine, as described in our previous study [15]. A tiny percutaneous needle biopsy, based on a semi-automatic needle, was used to obtain a small piece of skeletal muscle. Briefly, this muscle piece (about 15 mg) was immersed in a solution containing HAM’s F10 supplemented with 1% vol/vol Gentamicin (Euroclone S.p.A; Milan, Italy) and stored at 4 °C for 24 h. Subsequently, the procedure consisted of placing the small muscle piece in a sterile Petri dish and finely mincing it by adding a drop of Hyclone serum (Hyclone, Fisher Scientific Italia; Milan, Italy). It was obtained many sub-pieces of muscle, named explants. These were then placed on the surface of another Petri dish, spaced apart, and each explant was moistened with a drop (about 200 mL) of fetal bovine serum (FBS, Euroclone S.p.A; Milan, Italy). The Petri dishes containing the explants remained in a sterile incubator at 37 °C in a humidified atmosphere containing 5% CO_2_ for about two weeks, when numerous mononuclear cells, referred to as human muscle progenitor cells (hMPCs), migrated out of the explants and proliferated reaching 70% of confluence. Following detachment using trypsin–EDTA (Euroclone S.p.A; Milan, Italy), the cells were counted, and 180,000 cells were seated in a new Petri dish in growth medium containing (% vol/vol): HAM’s F10 (Euroclone S.p.A; Milan, Italy), 0.1 gentamycin and 1 penicillin/streptomycin 100X (Euroclone S.p.A; Milan, Italy), 20 FBS heat-inactivated (56° C, 36 min) (Hyclone, Fisher Scientific Italia; Milan, Italy), and 1 L-Glutamax 100× (Gibco, Fisher Scientific Italia; Milan, Italy). The hMPCs were passed once the cells reached about 70% confluence. The population doubling level (PDL) was determined at each passage using the formula: log_10_(N/n)/ln_2_, where *N* represents the number of cells at the time of passage and *n* the number initially plated. The cell population was considered to be at PDL 1 at the first passage. To sustain proliferation, hMPCs were cultured in growth medium (GM) composed of the following components (%vol/vol): HAM’s F10 (Euroclone S.p.A; Milan, Italy), 0.1% gentamycin, 1% penicillin/streptomycin 100X (Euroclone S.p.A; Milan, Italy), 20% heat-inactivated FBS (56 °C for 36 min) (Hyclone, Fisher Scientific Italia; Milan, Italy), and 1% L-Glutamax 100X (Gibco, Fisher Scientific Italia; Milan, Italy). This study was undertaken according to the Declaration of Helsinki and approved by the local ethics committee (G. D’Annunzio University—Chieti and Pescara; [12]).

### 2.3. RNA Isolation

To isolate high-quality total RNA, cells were treated with TRIzol^TM^ Reagent, following a specific protocol described by Thermo Fisher Scientific. The concentration of RNA was achieved with the Qubit RNA HS Assay Kit, and the RNA integrity and quality were assessed with the Qubit RNA IQ Assay Kit using a Qubit™ 4 Fluorometer (Thermo Fisher Scientific). The Qubit microRNA Assay Kits were also used to achieve a preliminary quantification and quality of small RNA in our samples.

### 2.4. miRNA Sequencing Library Preparation

For sequencing, 1 μg of total RNA was used to generate the miRNA sequencing library. RNAs were pooled together in each group to produce homogeneous sample material and to provide enough RNA for both the exploratory nature of sequencing and preliminary qPCR analyses [16,17]; however, this approach limits inter-individual variability assessment and is explicitly reported as a major limitation. Briefly, a pair of adaptors was sequentially ligated to the 3′ and 5′ ends of miRNA, and following ligation, a library was prepared by reverse transcription and PCR pre-amplification using TruSeq Small RNA Library Preparation Kits (Illumina; San Diego, CA, USA). The quality of the library was measured by the Qubit™ RNA Assay High Sensitivity Kits using Qubit 4.0 Fluorometer (ThermoFisher; Waltham, MA, USA). The final library (1 nM) was loaded into the Illumina iSeq100 system (Illumina), using a cartridge based on iSeq 100 i1 Reagent v2 chemistry (single read: 150 cycles; read length 1 × 75 bp). Raw sequence data (.bcl files) generated from Illumina iSeq100 system were converted into fastq files and demultiplexed by Local Run Manager (Illumina). Quality control check was verified (Table 1) using FastQC (Illumina). Illumina’s Bcl2fastq2 files were demultiplexed by Local Run Manager (Illumina), trimmed to remove adapter sequences using Cutadapt (Galaxy version v.4.4). Reads shorter than 18 bp and longer than 30 bp were discarded [18]. MiRNA sequences were aligned using CLC Genomics Workbench 24.0.1 (setting parameters: maximum mismatches 1, additional/missing bases 1) against the human genome (reference Illumina library). This app also performed the differential expression analysis between PRE and POST samples (normalization method: TMM; [19]). Differential expressions were calculated from the GLM, which corrects for differences in library size between the samples and the effects of confounding factors (Qiagen). A miRNA was considered differentially expressed when showing a log fold change (FC) of ≥1.5 (or ≤−1.5) between PRE and POST samples, with an FDR-adjusted *p*-value ≤ 0.05 [20].

### 2.5. Polyadenylation and cDNA Synthesis

The polyadenylation of RNA samples was carried out using the Poly(A) tailing of RNA kit by Applied Biological Materials (ABM; Richmond, BC, Canada). Briefly, RNA (100 ng), ATP 10 mM, Poly(A) polymerase, 5X Poly(A) polymerase reaction buffer, MnCl_2_ 25 mM, and nuclease-free H_2_O were added to a sterile tube sitting on ice, centrifuged, and incubated at 37 °C for 15 min. Then, the reaction was terminated by heating at 65 °C for 20 min in the thermocycler. cDNA synthesis was performed using the OneScript^®^ Hot Reverse Transcriptase (ABM). Briefly, the reaction was developed in a final volume of 20 μL containing universal poly-T-adapter primer (1 μM; GCGAGCACAGAATTAATACGACTCACTATAGGT_12_VN; Eurofins Genomics; Ebersberg, Germany), dNTPs, 5X RT Buffer, OneScript^®^ Hot RTase, nuclease-free H2O, and polyadenylated RNA. The tubes were incubated at 60 °C for 30 min and 85 °C for 5 min.

### 2.6. Real Time Quantitative PCR (qPCR)

To measure the expression of selected miRNAs on individual samples, qPCR was carried out on the QuantFinder^TM^ 48 Real-time Fluorescent Quantitative PCR Analyzer (Model BFQP-48) by BigFish Bio-tech (Hangzhou city, Zhejiang Province, China). The protocol was performed using 10 µL BlasTaq^TM^ 2X qPCR MasterMix (ABM) in a final volume of 20 µL containing 2 µL cDNA together with 1 µL forward (i.e., the mature miRNA sequence derived from the online database miRbase; 10 µM) and 1 µL reverse primer (i.e., the universal adapter sequence, 5′-GCGAGCACAGAATTAATACGAC-3′; 10 µM). Expression levels of miRNA in samples were normalized with respect to a stable housekeeping miRNA (i.e., miR-16-5p). The normalization strategy was selected based on the reported stability of miR-16-5p in skeletal muscle samples. qPCR validation was performed on individual samples to partially address the limitation of pooled RNA sequencing; however, due to the small sample size, inter-individual variability could not be fully characterized and represents a limitation of the study. The amplification reaction has been reached with the following cycle: 95 °C for 3 min for the enzyme activation, 15 s at 95 °C and 1 min at 60 °C, repeated 40 times. At the end of the amplification, a melting curve was performed for each reaction to discriminate among non-specific amplicons. To confirm qPCR products, a gel electrophoresis (1.8% agarose gel) method was used.

### 2.7. Statistical and miRNA Target Prediction Analyses

Data were expressed as mean ± standard deviation (SD) and a *t*-test was then used to compare miRNA expression values between PRE and POST groups. To investigate the pathways that could likely be regulated by the miRNAs whose expression changed in our samples, we used DIANA-mirPath v3.0 (https://dianalab.e-ce.uth.gr/html/mirpathv3/index.php?r=mirpath; accessed 12 November 2024). miRNA target-prediction was performed with multiple sources (i.e TargetScan −0.4 Context Score, microT-CDS 0.5 threshold, TarBase V7.0 FDR Correction, *p* < 0.05).

## 3. Results and Discussion

### 3.1. Sequencing and qPCR Results

During exposure to high-altitude regions, hypoxia-induced decline in physiological activities can be detected in muscle tissue [21]. Both oxygen delivery and blood dynamics respond to the combined stressors of hypoxia and physical exercise [14]. In skeletal muscle, O_2_ plays a pivotal role in both metabolism and the regulation of several intercellular pathways, which can modify proliferation, differentiation, and survival of cells within the myogenic lineage. In particular, a high-altitude induced impairment of the Vastus Lateralis muscle’s regenerative capacity was demonstrated [22]. MiRNAs are key regulators of these particular mechanisms, playing an essential role in the differentiation, maintenance, regeneration, and proper functioning of muscle cells. In fact, miRNAs regulate a complex network of gene expression to ensure the elderly progression and integrity of the myogenic program throughout various stages of muscle development or environmental stress, such as hypoxia, for a long period.

**Figure 2:**
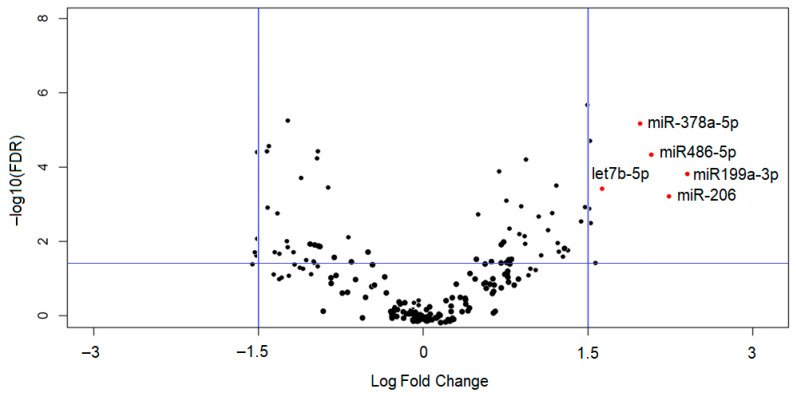
Volcano plot of differentially expressed mature miRNAs in PRE versus POST samples. Vertical lines indicate the threshold for a log fold change (FC) of 1.5 or −1.5-fold compared to the control (PRE group). The horizontal line represents the threshold of the FDR value. The red points lying in the top right sectors are significantly upregulated in POST versus PRE samples (FC ≥ 1.5). Statistical significance was determined using FDR-adjusted *p*-values (≤0.05).

Using a small RNA-seq profiling, we found that the expression level of several muscle-specific miRNAs changed (FC > 1.5, *p* < 0.05) after the “Lobuche Peak—Pyramid Exploration & Physiology” expedition, indicating that these hypoxia/muscular-derived miRNAs might participate in the physiological compensation process at high altitude. After sequencing, 1,065,606 reads were recognized as miRNA sequences (161 miRNAs; Figure 2).

Approximately 90% of the miRNA reads (∼960,000 reads) were concentrated on the 16 most abundant mature miRNAs. Among them, the miR-486-5p miRNA was the most abundant, representing approximately 80%. The results of the miRNAs global expression after correction for multiple tests (FDR < 0.05; *p*-value ≤0.05) in PRE and POST myocytes showed that five miRNAs were differentially expressed (Table 2).

**Figure 3:**
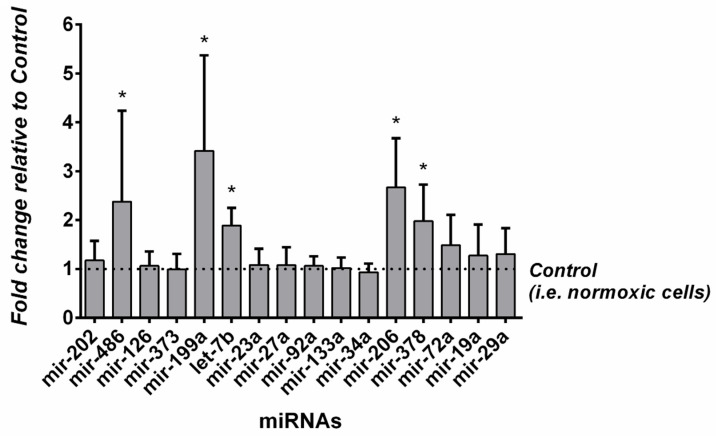
qPCR validation of selected miRNA expression in individual muscle biopsy samples collected before (PRE) and after (POST) the expedition. Data are expressed as fold change relative to PRE samples and presented as mean ± standard deviation (SD) (*n* = 6). Statistical significance was assessed using a paired Student’s *t*-test. * *p* < 0.05 vs PRE. miR-16-5p was used as an endogenous control.

Specifically, the miRNAs from the let family (let-7b-5p) and other miRNAs (miR-378a-5p, miR-486-5p, miR-199a-3p, miR-206) were upregulated in hypoxic myocytes with respect to the normal condition. These five differentially expressed miRNAs detected by NGS were validated by qPCR analysis, using miR-16-5 as a housekeeping miRNA. The results of qPCR confirmed that the levels of miR-199a-3p, let-7b-5p, miR-206, miR-378a-5p, and miR-486-5p were significantly (*p* < 0.05) increased in muscle biopsy samples from high-altitude trekkers collected immediately after the expedition (Figure 3). In hypoxic samples, miR-199a-3p and miR-206 were found to have an expression, respectively, greater than or equal to three times the level in the control (PRE), while the levels of let-7b-5p, miR-378a-5p, and miR-486-5p were detected to be ~2-fold expressed following the expedition. Also, other abundant mature miRNAs were analyzed by qPCR. The expression of these miRNAs (i.e., miR-202, miR-23a, miR-27a, miR-92a, miR-133a, miR-126, miR-373, miR-34, miR-72a, miR-518, miR-543, miR-19a, miR-29, miR-101, miR-232) did not change significantly in the hypoxic condition with respect to muscle biopsy samples collected before the expedition (Figure 3).

The data regarding the high levels of miR-486-5p expression compared to other miRNAs should not be surprising since it has been shown that miR-486 is highly expressed in skeletal muscle tissue and it is considered a muscle-enriched miRNA that plays a key role in maintaining muscle homeostasis and functionality [11]. Studies examining miR-486 in the context of muscular hypoxia aim to elucidate its specific regulatory mechanisms within skeletal muscle cells, especially concerning mitochondrial function, vascularization, and adaptations that facilitate muscle endurance and performance in low-oxygen environments [23,24]. Another significantly regulated miRNA belongs to the myomiRNAs family (i.e., miR-206) while miR-199, let-7b, and miR-378 can be classified as hypoxamiRNAs [4,25]. During myogenesis, miR-206 is currently known to be the best muscle tissue-specific miRNA and is involved in myogenic processes since it is exclusively expressed in skeletal muscle cells [26]. A study by Dey et al. [27] demonstrated that differentiation and proliferation of myoblasts were modulated by miR-206 and miR-486 through the reduction of their direct target, the Paired-box (PAX) 7, by directly targeting its 3′ untranslated region (UTR). Overexpression of either of these miRNAs in myoblasts accelerates differentiation [27]. Nakasa et al. [28] demonstrated that local injection of miR-206 mixture in rat skeletal muscle increased muscle regeneration via increased myogenic regulatory factors such as myogenin. Furthermore, upregulation of muscular miRNAs, such as miR-206, might be linked to the reduction of muscle inflammation [29]. All these studies confirm that miR-206 holds significant importance in regulating skeletal physiology, from myogenesis to metabolism and inflammation control. In this regard, hypoxamiRNAs are a group of miRNAs that are upregulated or downregulated in response to low oxygen levels, influencing various myocyte responses to hypoxia. miR-378 has been identified as a hypoxamiRNA due to its responsiveness to low O_2_ levels in various cellular contexts [30]. In hypoxic conditions, miR-378 expression has been observed to increase, contributing to the cellular adaptation to low oxygen environments, promoting cell survival and vascularization in mesenchymal stem cells [25]. High levels of miR-378 can increase cell survival mechanisms under hypoxia by controlling genes associated with apoptosis and angiogenesis [31]. Among hypoxia-related regulatory networks, the thrombospondin-1 (TSP1)/CD47 axis represents a relevant anti-angiogenic pathway modulated by both HIF and miRNAs. Notably, miR-378a-5p has been reported to regulate CD47, suggesting a possible intersection between hypoxia signaling, vascular adaptation, and cAMP-related pathways. Similarly, miR-199 has been implicated in the regulation of myogenesis from precursor muscle cells [32]. miR-199 influences the differentiation of myoblasts into mature muscle cells, regulating muscle-specific processes, including myogenic regulatory factors (MRFs) such as MyoD and myogenin, as well as other genes associated with muscle cell function and contractility. Furthermore, miR-199a-5p is classified as a hypoxia-sensitive miRNA and serves as an important regulator in cardiovascular pathophysiology. In fact, miR-199a-5p was positively associated with exercise capacity during chronic hypoxia at high altitudes [21].

### 3.2. Analysis and Clustering of the Pathways Regulated by Myo/Hypoxia-miRNAs

**Figure 4:**
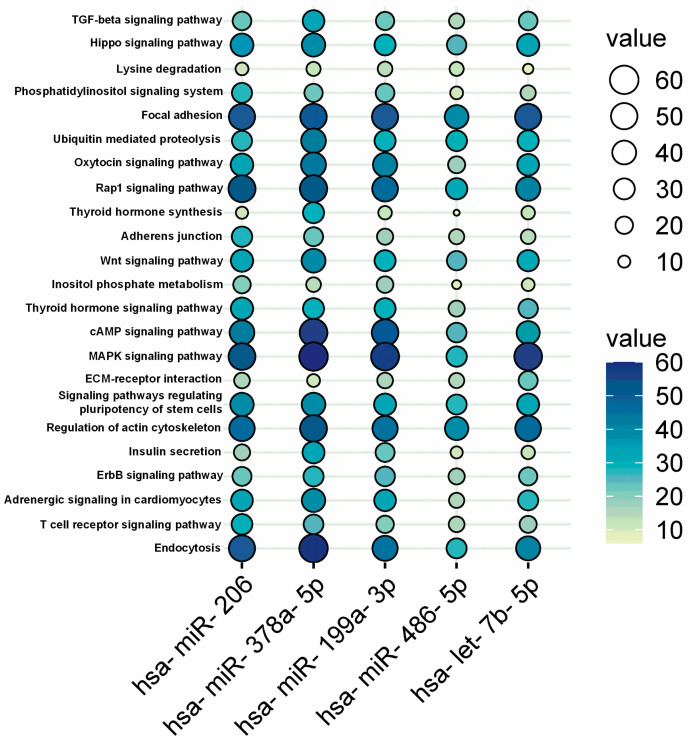
The prediction of potential pathways regulated by the differentially expressed miRNAs. The color and size of the circles indicate the reliability of the prediction results. The names of the differentially expressed miRNAs are on the bottom, while the potentially regulated pathways are on the left (DIANA-mirPath v3 analysis). Enrichment significance was calculated using adjusted *p*-values (FDR correction).

In order to identify the specific pathways regulated by the miRNAs selected for qPCR validation (i.e., miR-206, miR-486-5p, miR-199a-3p, let-7b-5p, miR-378a-5p), we performed enrichment analysis of the genes targeted by those miRNAs. Applying this framework, the current study is the first to identify the most relevant targets and to provide a validation on a very specific cohort of athletes (i.e., high-altitude trekkers). In enrichment analysis, we found several connections between miRNAs and pathways that may play a role in physiological regeneration or differentiation in muscle cells. Among many other associated functions, focal adhesion (*p*-value = 0.001), regulation of actin cytoskeleton (*p*-value = 0.026), and Rap-1 (*p*-value = 0.007), cAMP (*p*-value = 0.017), MAPK (*p*-value = 0.019), and Hippo (*p*-value ≤ 0.001) signaling pathways were predicted were predicted as potential targets of the differentially expressed miRNAs (Figure 4), based on in silico enrichment analysis Importantly, these pathway associations are based on predictive bioinformatic analyses and do not imply direct functional activation or causal regulation.

**Figure 5:**
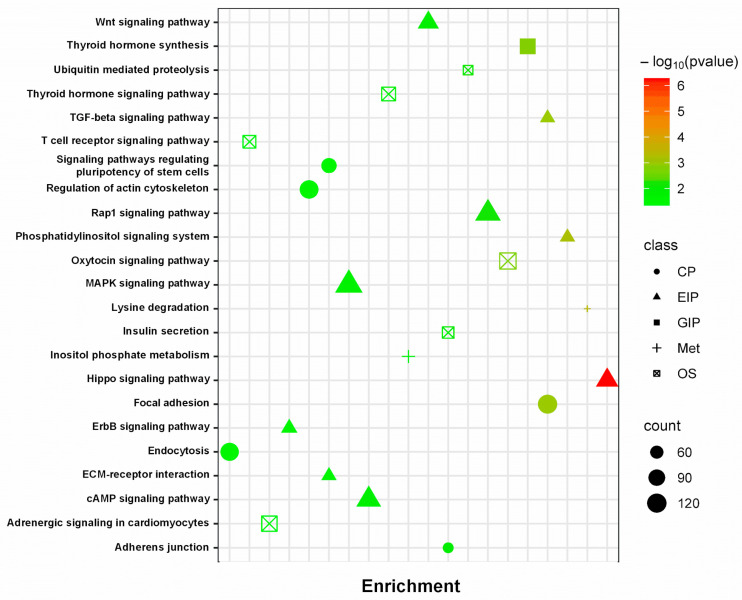
KEGG enrichment analysis for pathway-based clustering based on the number of target genes associated with the hypoxia condition. Grouping classes are Cellular Processes (CPs), Environmental Information Processing (EIP), Genetic Information Processing (GIP), Metabolism (MET), and Organismal Systems (OSs). Enrichment significance was calculated using adjusted *p*-values (FDR correction).

For a better understanding of the process types that these miRNAs might be able to regulate, a further analysis was performed to cluster the regulated pathways in categories (Figure 5).

Specifically, many of the target genes are related to specific KEGG map processes such as Environmental Information Processing (EIP), Cellular Processes (CPs), and Organismal Systems (OSs). In this regard, the highest number of controlled genes by selected miRNAs belongs to the EIP group (n° targeted genes = 815), followed by CP (n° targeted genes = 518) and OS (n° targeted genes = 426) groups. The EIP group encloses all the pathways that share similar or related functions, such as cell growth and proliferation in response to growth factors or stress signals (MAPK, Hippo, Rap-1, cAMP signaling pathways). It has been evaluated that hypoxia can impact the Hippo pathway by influencing the activity of its downstream effectors involved in maintaining cell survival, reducing the transcription of apoptotic genes [33]. Hypoxia might affect Rap-1 signaling by influencing cell adhesion and migration processes [34], potentially altering integrin-mediated signaling in response to oxygen deprivation. However, cAMP and MAPK signaling pathways are highly responsive to hypoxia since these signaling pathways undergo adjustments in their activities and target genes to facilitate cellular adaptations under low oxygen conditions. One of the central connections between the MAPK pathway and hypoxia is through the regulation of HIF-1, a key transcription factor that modulates cellular responses to low O_2_ levels [35]. The CP group is constituted by interconnected cellular pathways (e.g., focal adhesion and regulation of actin cytoskeleton) that work together to facilitate cell adhesion, migration, and structural organization. Hypoxia induces various changes in the behavior of cells, such as composition and turnover, affecting their ability to transmit mechanical signals between cells and the extracellular environment [36]. In addition, chronic low levels of O_2_ influence the actin cytoskeleton by modulating the activity of Rho GTPase and actin-binding proteins, leading to the reduction of cytoskeletal organization and dynamics (e.g., actin polymerization, stress fiber formation) [37]. Hypoxia can indeed modulate the endocrine system, including the synthesis and function of hormones, such as thyroid hormones [38]. This particular regulation is highlighted by the regulation of genes belonging to the OS group that are potential targets of miRNAs analyzed in our study. After enrichment analysis, it has been observed that the OS group encloses thyroid hormone, oxytocin signaling pathways, and insulin secretion. In this regard, the specific effect on these pathways seems to be mediated by the modulation of HIF-1, as also reported in other studies [38,39]. Nevertheless, the effects of hypoxia on endocrine systems highlight the complexity of cellular responses to O_2_ deprivation, impacting different physiological functions and regulatory systems. These findings should also be interpreted within a broader physiological framework. High-altitude exposure induces systemic adaptations involving oxygen transport, aerobic capacity (VO_2_), and endocrine regulation, which are tightly interconnected with skeletal muscle function. The miRNA changes observed in this study may therefore reflect not only local molecular responses but also integrated physiological adjustments involving metabolic, hormonal, and circulatory systems. In particular, hypoxia-induced endocrine responses and systemic stress signaling may contribute to the regulation of muscle remodeling and adaptation, highlighting the need to interpret miRNA modulation within a multi-level physiological context rather than as an isolated molecular phenomenon. Acute exercise leads to a rapid and transient miRNA response, both in systemic circulation [40] and muscle tissue [41]. The effect of hypoxia on miRNA expression starts early after exposure and persists over a longer duration or exhibits a delayed response; this time-course of response is influenced differentially by HIF-1 and HIF-2 and is linked to diverse miRNAs [42]. Confounding factors, including physical activity, nutritional status, HPA axis activation, and acetazolamide use, may have contributed to the observed miRNA changes and should be considered as potential sources of variability when interpreting the results [43]. Here, we report preliminary evidence from miRNome analysis indicating that several myomiRNAs and hypoxamiRNAs are upregulated as a late molecular response to the combined stress of prolonged trekking and hypoxic exposure.

This study has several important limitations. First, the small sample size (*n* = 6) and the use of pooled RNA samples significantly limit statistical robustness and prevent assessment of inter-individual variability. Second, the absence of a control group (e.g., exercise under normoxic conditions) precludes the discrimination between the effects of hypoxia, physical activity, and pharmacological intervention. Third, biopsies were performed after returning from the expedition, introducing potential re-adaptation effects. Additional confounding factors, including nutritional status, endocrine responses (e.g., HPA axis activation), and acetazolamide use, may have influenced miRNA expression. Furthermore, this study relies on differential expression and in silico pathway prediction without functional validation, limiting mechanistic interpretation. Therefore, the present findings should be considered exploratory and hypothesis-generating rather than conclusive.

## 4. Conclusions

This study provides an exploratory characterization of skeletal muscle miRNA expression changes associated with high-altitude trekking, highlighting molecular responses emerging from the combined exposure to hypoxia and prolonged physical activity. The observed modulation of muscle-enriched and hypoxia-responsive miRNAs supports the existence of a complex regulatory network linking environmental stress to cellular pathways involved in muscle adaptation. The current results are based on a mixed-sex cohort and reveal consistent miRNA expression trends. While sex-stratified analyses were underpowered, the observed patterns support the need for future studies specifically addressing sex-dependent effects. By identifying miRNA expression patterns and predicted target pathways related to cytoskeletal remodeling, signaling, and tissue regeneration, this work contributes to a deeper understanding of human physiological adaptation to extreme environments. Importantly, the miRNAs identified in this study may provide potential molecular clues for evaluating physical endurance and adaptation at high altitudes; however, their utility as reference indicators requires validation through future large-scale studies integrating direct correlations with functional and performance-related outcomes. Further investigations addressing the temporal dynamics of miRNA expression, their relationship with hypoxia-responsive signaling, and the regulation of downstream target genes will be essential to clarify the mechanisms coordinating muscle adaptation to hypoxic stress.

## Figures and Tables

**Table 1:** FastQC quality checks on the total raw sequence data.

Total Cluster	%PF	% ≥ Q30	Sample	Illumina Index	Input Reads	Reads with Adapters	Read Passed (>18 and <30 bp)
3,465,412	42.8	80.4	PRE	RPI10	1,196,156	1,085,453	576,185
			POST	RPI12	987,662	903,365	489,421

Q30: Quality score, %PF: Cluster Passing Filter.

**Table 2:** miRNAs significantly differentially expressed in muscle biopsy samples from high-altitude trekkers, collected before and immediately after the “Lobuche Peak—Pyramid Exploration & Physiology” expedition.

miRNA	Log Fold Change	Expression Trend	*p*-Value
*hsa-miR486-5p*	1.98	Up	1.0 × 10^−3^
*hsa-miR-199a-3p*	2.42	Up	1.2 × 10^−4^
*hsa-let7b-5p*	1.55	Up	1.0 × 10^−4^
*hsa-miR-206*	2.12	Up	1.6 × 10^−4^
*hsa-miR-378a-5p*	1.74	Up	1.7 × 10^−4^

## Data Availability

The data that support the findings of this study are available in the Section 2 and Section 3 of this article. Additional data are available on request from the corresponding authors.
